# The MICA-129Met/Val dimorphism affects plasma membrane expression and shedding of the NKG2D ligand MICA

**DOI:** 10.1007/s00251-015-0884-8

**Published:** 2015-11-19

**Authors:** Antje Isernhagen, Daniela Schilling, Sebastian Monecke, Pranali Shah, Leslie Elsner, Lutz Walter, Gabriele Multhoff, Ralf Dressel

**Affiliations:** Institute of Cellular and Molecular Immunology, University Medical Center Göttingen, Humboldtallee 34, 37073 Göttingen, Germany; Department of Radiation Oncology, Klinikum rechts der Isar, Technische Universität München (TUM), Munich, Germany; Institute of Innovative Radiotherapy (iRT), Radiation Immune Biology, Department of Radiation Sciences (DRS), Helmholtz Zentrum München, Munich, Germany; Primate Genetics Laboratory, German Primate Center, Göttingen, Germany; DZHK (German Center for Cardiovascular Research), Partner site Göttingen, Göttingen, Germany

**Keywords:** Single nucleotide polymorphism, Major histocompatibility complex (MHC) class I chain-related molecules A (MICA), Plasma membrane expression, Proteolytic shedding, Tumor cells

## Abstract

The MHC class I chain-related molecule A (MICA) is a ligand for the activating natural killer (NK) cell receptor NKG2D. A polymorphism causing a valine to methionine exchange at position 129 affects binding to NKG2D, cytotoxicity, interferon-γ release by NK cells and activation of CD8^+^ T cells. It is known that tumors can escape NKG2D-mediated immune surveillance by proteolytic shedding of MICA. Therefore, we investigated whether this polymorphism affects plasma membrane expression (pmMICA) and shedding of MICA. Expression of pmMICA was higher in a panel of tumor (*n* = 16, *P* = 0.0699) and melanoma cell lines (*n* = 13, *P* = 0.0429) carrying the *MICA-129Val/Val* genotype. *MICA-129Val* homozygous melanoma cell lines released more soluble MICA (sMICA) by shedding (*P* = 0.0015). MICA-129Met or MICA-129Val isoforms differing only in this amino acid were expressed in the MICA-negative melanoma cell line Malme, and clones with similar pmMICA expression intensity were selected. The MICA-129Met clones released more sMICA (*P* = 0.0006), and a higher proportion of the MICA-129Met than the MICA-129Val variant was retained in intracellular compartments (*P* = 0.0199). The MICA-129Met clones also expressed more *MICA* messenger RNA (*P* = 0.0047). The latter phenotype was also observed in mouse L cells transfected with the MICA expression constructs (*P* = 0.0212). In conclusion, the MICA-129Met/Val dimorphism affects the expression density of MICA on the plasma membrane. More of the MICA-129Met variants were retained intracellularly. If expressed at the cell surface, the MICA-129Met isoform was more susceptible to shedding. Both processes appear to limit the cell surface expression of MICA-129Met variants that have a high binding avidity to NKG2D.

## Introduction

The activity of natural killer (NK) cells is controlled by activating and inhibitory natural killer receptors. NKG2D (NK group 2, member D) is an activating receptor, which is expressed mainly on NK cells and CD8^+^ αβT cells (Champsaur and Lanier 2010; Raulet et al. 2013). NKG2D signaling triggers cytotoxicity (Billadeau et al. 2003) and cytokine secretion of NK cells (Andre et al. 2004), whereas it functions as a co-stimulatory molecule on CD8^+^ αβT cells (Groh et al. 2001). NKG2D-mediated pathways are important for the elimination of malignant cells (Guerra et al. 2008) and for defense against some pathogens (Fang et al. 2008; Wesselkamper et al. 2008). The NKG2D receptor recognizes several ligands on target cells, which include the major histocompatibility complex (MHC) class I chain-related molecules A (MICA) and B (MICB) and the six members of the UL16-binding proteins (ULBP1-6) (Chitadze et al. 2013; Choy and Phipps 2010).

*MICA* and *MICB* are encoded in the HLA complex (Bahram et al. 1994; Choy and Phipps 2010; Leelayuwat et al. 1994), and *MICA* is the most polymorphic non-classical class I gene (http://www.ebi.ac.uk/imgt/hla/). The domain structure of MICA is similar to classical class I molecules with three extracellular domains (α1, α2, and α3), a transmembrane segment, and a carboxy-terminal cytoplasmic tail. However, MICA is not associated with β2-microglobulin and does not present peptides. MICA is constitutively expressed on a few cell types, including gastrointestinal epithelium (Groh et al. 1996); however, following cellular or genotoxic stress (Gasser et al. 2005; Groh et al. 1996), it can be induced on malignant or virus-infected cells (Champsaur and Lanier 2010; Raulet et al. 2013).

Proteolytic shedding of MICA can result in a tumor immune escape mediated by immunosuppressive soluble MICA (sMICA) (Chitadze et al. 2013; Groh et al. 2002; Salih et al. 2002). Soluble MICA can induce NKG2D downregulation by rapid endocytosis and partial lysosomal degradation resulting in the impairment of NK cell cytotoxicity (Roda-Navarro and Reyburn 2009) and co-stimulation of CD8^+^ T cells via NKG2D. MICA is cleaved at the cell surface by members of the family of matrix metalloproteases (MMPs) and the “a disintegrin and metalloproteinase” (ADAM) family, including ADAM10 and ADAM17 (Groh et al. 2002; Kaiser et al. 2007; Salih et al. 2002; Waldhauer et al. 2008). The α3 domain of MICA forms a complex with the disulphide isomerase/chaperon endoplasmic reticulum protein 5 (ERp5) on the surface of tumor cells, which induces a conformational change enabling the proteolytic cleavage of MICA. Shedding of NKG2D ligands has been reported for many cancers and some hematopoietic malignancies (Chitadze et al. 2013). Not only sMICA but also tumor-derived exosomes, which contain MICA (Clayton et al. 2008), may contribute to a downregulation of NKG2D. A number of clinical studies showed an association between tumor-associated or soluble NKG2D ligands and disease progression and poor prognosis in different malignant diseases (El-Gazzar et al. 2013). Taken together, these tumor-mediated counter-regulation mechanisms appear to contribute to tumor evasion from NK cell and CD8^+^ T cell-mediated immunity.

Several *MICA* polymorphisms have been reported to affect MICA shedding including a single nucleotide polymorphism (SNP) in the promoter region, a microsatellite in exon 5 encoding the transmembrane region, and the MICA-129Met/Val dimorphism in α2 domain of the MICA protein.

The SNP at -1878 (rs2596542) in the promoter region of the *MICA* gene was found to be associated with the risks of hepatitis C (Kumar et al. 2011) and hepatitis B virus-induced hepatocellular carcinoma (Kumar et al. 2012; Tong et al. 2013). In all three studies, an association of higher sMICA serum levels with the *G* allele was observed. The *G* allele was found to have a higher transcriptional activity (Lo et al. 2013), which might explain the effects on sMICA serum levels indirectly by higher MICA expression intensities.

The transmembrane region of MICA, encoded in exon 5, contains a polymorphic microsatellite, which varies in the number (4 to 9) of alanine encoding *GCT* repeats (Fodil et al. 1996; Mizuki et al. 1997; Ota et al. 1997). The *MICA-A5.1* polymorphism contains five triplet repeats plus one additional insertion (**G**GCT/**A**GCC) causing a frame shift, which results in a premature stop codon in the transmembrane region. *MICA* alleles containing the *MICA-A5.1* variant, such as MICA*008, have a number of unique features including recruitment of the protein to exosomes, which might be explained by acquisition of a GPI (glycosylphosphatidylinositol) anchor by this modification that replaces the transmembrane domain (Ashiru et al. 2013). The *MICA-A5.1* polymorphism has been associated with autoimmune diseases (Fojtikova et al. 2011; Lü et al. 2009; Novota et al. 2005; Triolo et al. 2009), the risk of cytomegalovirus reactivation in HIV-1-infected patients (Moenkemeyer et al. 2009), and several malignancies (Chen et al. 2013; Jiang et al. 2011; Lavado-Valenzuela et al. 2009; Luo et al. 2011; Tamaki et al. 2007; Tian et al. 2006; Tong et al. 2013). Moreover, donor *MICA A5.1* genotype and anti-MICA sensitization was identified as a risk factor for kidney transplant survival (Tonnerre et al. 2013). In patients with oral squamous cancer (Tamaki et al. 2009) and in patients with hepatocellular carcinoma (Jiang et al. 2011), the *A5.1* genotype was associated with higher sMICA serum levels, and Raji cells constructed to express the *MICA A5.1* allele produced more sMICA than cells transfected to express a full-length *MICA A5* allele (Lü et al. 2009).

The SNP (rs1051792) at nucleotide position 454 (*G*/*A*) of the *MICA* gene, which leads to an amino acid substitution from valine (Val) to methionine (Met) at position 129 in the α2 domain of the MICA protein, has been described to affect NKG2D binding avidity (Steinle et al. 2001). This SNP has been associated with the risk of nasopharyngeal carcinoma (Douik et al. 2009), hepatitis B virus-induced hepatocellular carcinoma (Tong et al. 2013), chronic (Boukouaci et al. 2009) and acute graft versus host disease (Isernhagen et al. 2015), the risk of ventricular systolic dysfunction in chronic Chargas heart disease (Ayo et al. 2015), and a number of autoimmune diseases, including ankylosing spondylitis (Amroun et al. 2005), rheumatoid arthritis (Kirsten et al. 2009), inflammatory bowel disease (Lopez-Hernandez et al. 2010; Zhao et al. 2011), lupus erythematosus (Yoshida et al. 2011), type I diabetes (Raache et al. 2012), and psoriatic disease (Pollock et al. 2013). In patients with ulcerative colitis, the *MICA-129Val/Val* genotype was associated with higher sMICA serum levels (Zhao et al. 2011) and the *MICA-129Met* allele was associated with lower sMICA serum levels in hepatitis B virus-induced hepatocellular carcinoma patients and healthy controls (Tong et al. 2013).

Tong and colleagues correlated 10 *MICA* polymorphisms with sMICA serum levels in hepatitis B virus-induced hepatocellular carcinoma patients (Tong et al. 2013). In addition to associations mentioned before, they found significantly higher sMICA serum levels associated with the coding variants *MICA-175Ser* and *MICA-251Arg* and the microsatellite variants *A4* and *A9* (Tong et al. 2013).

We recently described that the high-avidity MICA-129Met variant is characterized by stronger and faster NKG2D signaling, triggering of more NK cell cytotoxicity and interferon-γ release, a rapid co-stimulation of CD8^+^ T cells but also a rapid downregulation of NKG2D (Isernhagen et al. 2015). Therefore, we compared herein the MICA-129Met and MICA-129Val variants also with respect to MICA expression and shedding.

## Methods

### Cell culture and transfection

The human tumor cell lines H1339, EPLC-272H, A549 (lung), FaDu, SAS, Cal33, BHY, UT15 (squamous cell carcinoma of the head and neck), CX2, HCT116 (colon), Panc-1 (pancreas), MCF-7, T47D, MDA-MB-231 (breast), and Hela (cervix) were cultured in Roswell Park Memorial Institute (RPMI) 1640 medium (Sigma-Aldrich, Taufkirchen, Germany) supplemented with 10 % fetal calf serum (FCS) (Sigma-Aldrich), 2 mM l-glutamine, 1 mM sodium pyruvate 100 IU/ml penicillin, and 100 μg/ml streptomycin. Human melanoma cell lines (Dressel et al. 1998) and mouse fibroblast L cells were maintained in NaHCO_3_-buffered Dulbecco’s modified Eagle medium (DMEM) supplemented with 10 % FCS (Biochrom, Berlin, Germany), 2 mM l-glutamine, 1 mM sodium pyruvate, 50 μM 2-mercaptoethanol, 100 U/ml penicillin, and 100 μg/ml streptomycin. Cell culture plastic materials were from Greiner (Frickenhausen, Germany) or Sarstedt (Nümbrecht, Germany). To induce MICA expression, the melanoma cells were cultured in DMEM with 10 μM of the histone deacetylase (HDAC) inhibitor suberoylanilide hydroxyamic acid (SAHA) (Qbiogene-Alexis, Grünberg, Germany) 20 h before being used for experiments. The pCMV6-AC-MICA-129Met or pCMV6-AC-MICA-129Val expression constructs and the L-MICA-129Met and L-MICA-129Val cells have been described previously (Isernhagen et al. 2015). Malme cells were transfected with 50 μg of *Pvu*I-linearized constructs by electroporation. After selection (1 mg/ml G418, Biochrom, Berlin, Germany), clones (Malme-MICA-129Met and Malme-MICA-129Val) were obtained by limiting dilution.

### Genotyping

One to five million cells were harvested, washed with phosphate-buffered saline (PBS), resuspended in 500 μl lysis buffer (100 mM NaCl, 50 mM EDTA (pH 8.0), 10 mM Tris–HCl (pH 8.0), 0.5 % sodium dodecyl sulfate, 0.1 mg/ml proteinase K, 20 μg/ml RNase A), and incubated at 50 °C shaking at 500 rounds per minute (rpm) overnight. Cell lysis was followed by phenol-chloroform extraction and alcohol precipitation using 2 volumes 100 % ethanol and 1/10 volume 5 M lithium chloride. Genomic DNA was dissolved in dH_2_O or 8 mM NaOH and stored at 4 °C. The SNP rs1051792 (*G*/*A*) leading to a substitution of Val (*G*) by Met (*A*) at position 129 of MICA was genotyped by a TaqMan assay (Applied Biosystems, Foster City, CA, USA) containing the forward primer 5′-GCTCTTCCTCTCCCAAAACCT-3′ and the reverse primer 5′-CGTTCATGGCCAAGGTCTGA-3′ and the two allele-specific dye-labeled probes FAM-5′-AATGGACAGTGCCCC-3′ and VIC-5′-AATGGACAATGCCCC-3′. Results were confirmed by Sanger sequencing, if required.

### Flow cytometry

Flow cytometry was performed with a FACSCalibur™ flow cytometer and CellQuestPro™ software (BD Biosciences, Heidelberg, Germany). Cell surface expression of MICA on propidium iodide negative melanoma and mouse L cells was examined using the anti-MICA monoclonal antibody (mAb) AMO1 (Bamomab, Gräfelfing, Germany); 10^6^ cells were incubated with 1 μg mAb in 100 μl PBS for 45 min. After washing with PBS, 1 μl of the fluorescein isothiocyanate (FITC)-conjugated goat anti-mouse IgG Ab (155-095-062, Jackson Laboratories, via Dianova, Hamburg, Germany) was applied in 100 μl PBS for 30 min as secondary reagent. MICA expression on all other human tumor cells was determined with an allophycocyanin (APC)-conjugated MICA antibody (clone 159227, mouse IgG_2b_ R&D Systems, Wiesbaden, Germany) compared to an isotype-matched control antibody. All stainings were done at 4 °C in the dark.

### Confocal microscopy

Confocal microscopy was performed with Malme cells that were grown to sub-confluence (70 to 80 %) on acid-washed glass coverslips. After washing with PBS, cells were fixed and permeabilized with ice-cold methanol acetone solution (7:3) for 15 min at −20 °C. Cells were air dried and subsequently incubated with a blocking solution (0.5 % bovine serum albumin in PBS) for 1 h at room temperature. Subsequently, the cells were covered with 50 μl of the primary antibody anti-MICA (AMO1; 1 mg/ml) diluted in 1:50 blocking solution, and incubated overnight at 4 °C in a humidified atmosphere. After washing five times with PBS, 1 μl of the secondary Cy2-conjugated goat anti-mouse antibody (115-225-062, Jackson Laboratories, via Dianova, Hamburg, Germany) diluted in 100 μl blocking solution plus 1 μl of Hoechst 33342 (10 mg/ml) was added to the cells before incubation for 1 hour at room temperature. The coverslips were washed five times with PBS before they were mounted on microscope slides using fluorescence mounting medium (Dako, Hamburg, Germany). Microscopy was performed with the Zeiss LSM 510 Axioplan 2 confocal microscope.

### Enzyme-linked immunosorbent assay (ELISA)

Concentrations of sMICA in the supernatants of cells (10^6^ cells, 10 ml medium, 24 h) and of intracytoplasmic (ic) MICA in cell lysates in relation to the total protein content were determined using the human MICA DuoSet (R&D Systems). These assays were performed according to the manufacturer’s protocols. All samples were analyzed in duplicate in comparison to a standard curve of MICA.

### Sodium dodecyl sulfate polyacrylamide gel electrophoresis (SDS-PAGE) and immunoblotting

For Western blot analysis, the cells were lysed in 25 μl Nonidet P-40 buffer (1 %) before 25 μl reducing loading buffer was added. After incubation for 4 min at 95 °C, the lysates were loaded on 10 % SDS gels for electrophoresis at 40 to 100 V for approximately 3 h. Then the proteins were blotted onto a nitrocellulose membrane (Roth, Karlsruhe, Germany) for 1 h using a semi-dry blotting technique (1 mA/cm^2^). The membrane was blocked in Tris-buffered saline with 0.1 % Tween-20 (TBS-T) with 5 % (*w*/*v*) non-fat dry milk for 1 h, washed, and then incubated with a biotinylated anti-MICA Ab (0.4 μg/ml, polyclonal goat IgG, BAF1300, R&D Systems) and an anti-β-actin mAb (1:10,000, mouse IgG_1_, clone AC-15, Sigma-Aldrich) in TBS-T together with 5 % (*w*/*v*) non-fat dry milk overnight at 4 °C. After being washed three times for 10 min in TBS-T, the membrane was incubated with horseradish peroxidase (HRP)-conjugated streptavidin (1:2000, BioLegend, Fell, Germany) and HRP-labeled goat-anti-mouse IgG secondary Ab (1:10,000, 115-035-003, Jackson Laboratories, via Dianova, Hamburg, Germany). Detection was done using an enhanced chemiluminescence (ECL) kit (GE Healthcare), and chemiluminescence was measured using a digital image acquisition system (Intas Chemilux Entry, Intas, Göttingen, Germany).

### Quantitative polymerase chain reaction (qPCR)

Total RNA extraction and complementary DNA (cDNA) synthesis were carried out as described previously (Dressel et al. 2009). For *MICA*, the following forward and reverse primers were generated (5′- ACT TGA CAG GGA ACG GAA AGG A -3′ and 5′- CCA TCG TAG TAG AAA TGC TGG GA -3′). The messenger RNA (mRNA) expression of the housekeeping gene glyceraldehyde 3-phosphate dehydrogenase (*GAPDH*) (5′- ACG AAT TTG GCT ACA GCA ACA GGG -3′ and 5′- TCT ACA TGG CAA CTG TGA GGA GG -3′) for human cell lines or hypoxanthine guanine phosphoribosyl transferase 1 (*Hprt1*) (5′- GTC CTG TGG CCA TCT GCC TA- 3′ and 5′- GGG ACG CAG CAA CTG ACA TT- 3′) for mouse L cells were always monitored as internal control. Amplification reactions were carried out in 96-well plates in 25 μl reaction volumes with the Power SYBR® green PCR master mix (Applied Biosystems, Foster City, USA). The PCR reaction plates were preheated for 2 min at 50 °C and for 10 min at 95 °C followed by 40 cycles of denaturation (15 s at 95 °C) and amplification (1 min at 60 °C). All reactions were performed in technical triplicates using an ABI 7500 Real-Time PCR System. For the data analysis, the ABI 7500 SDS software (Applied Biosystems) was used. The variations in cDNA concentration in different samples were normalized to the housekeeping genes *GAPDH* or *Hprt1*. The cycle threshold (ct) values obtained for the target gene (tg), i.e., *MICA*, were corrected by the ct value obtained for the housekeeping gene (hkg) in the same sample. The relative amount of transcripts was then expressed as Δct value (ct for hkg minus ct for tg).

### Statistics

The data were evaluated with SPSS (IBM, Ehningen, Germany) or WinStat software (R. Fitch Software, Bad Krozingen, Germany). Pearson correlation and *t* tests were employed after confirming normal distribution of the data.

## Results

### Correlation of MICA expression and shedding in various tumor and melanoma cell lines with the *MICA-129* genotype

**Fig. 1:**
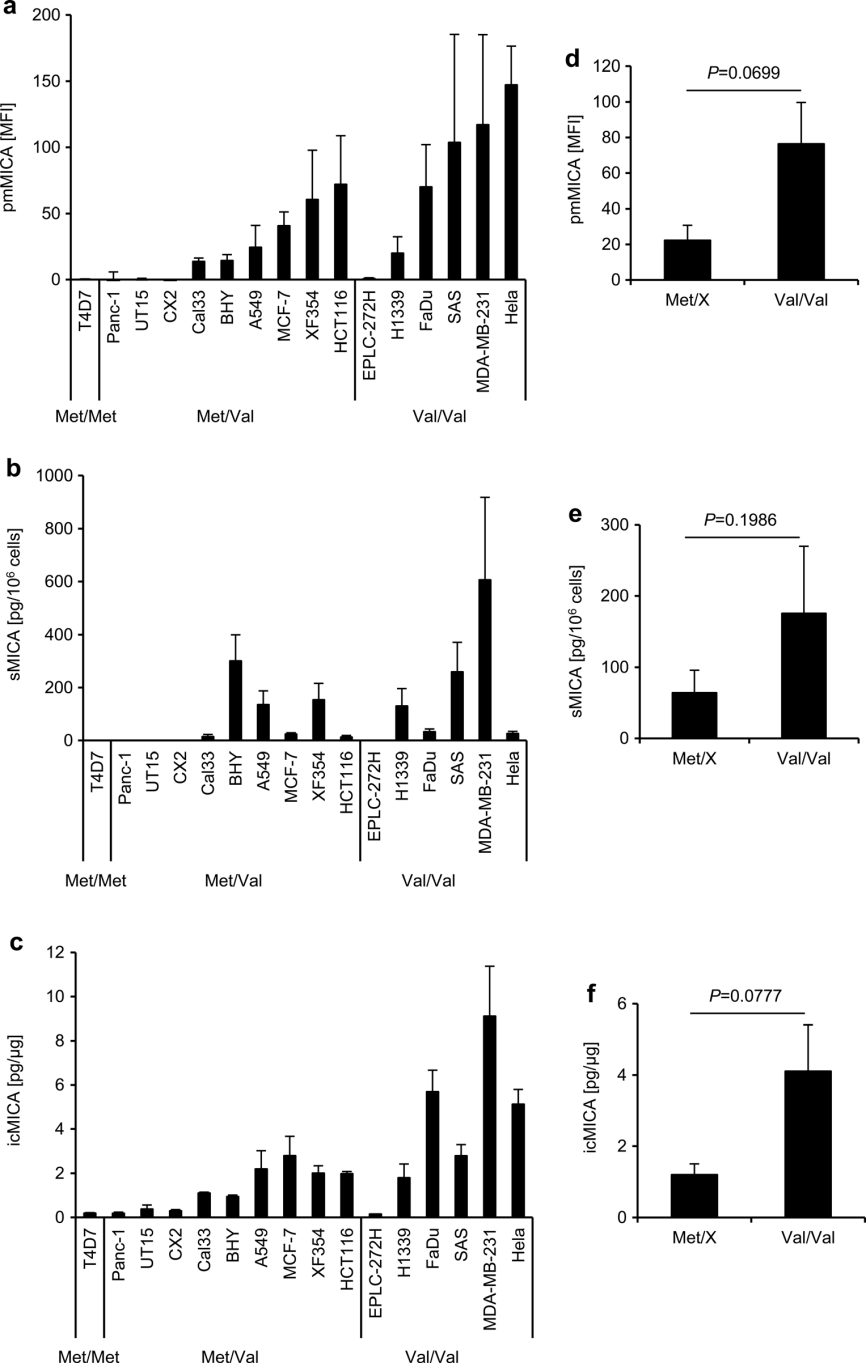
MICA expression on the plasma membrane and release of sMICA in human tumor cell lines with different *MICA-129* genotypes. **a** The expression of pmMICA on human tumor cell lines carrying the different *MICA-129* genotypes (Met/Met, Met/Val, and Val/Val) was analyzed by flow cytometry. The mean fluorescence intensities (*MFI*) of pmMICA are displayed as means plus standard deviation (*SD*) (*n* ≥ 5). **b** In parallel, the amounts of soluble MICA (*sMICA*) in the supernatants were determined by ELISA and are shown as means plus SD of sMICA (pg/10^6^ cells). **c** The intracellular MICA (*icMICA*) was determined by ELISA and is shown as means plus SD. **d**–**f** The data were grouped according to the *MICA-129* genotype, and cell lines carrying one or two *MICA-129Met* alleles (Met/X, i.e., Met/Met and Met/Val) were compared to cell lines which were homozygous for the *MICA-129Val* allele. The data are shown as means plus standard error of the mean (*SEM*) and they were analyzed by *t* tests

We analyzed the expression of MICA on the plasma membrane (pmMICA) in a panel of 16 human tumor cell lines of different entities (Fig. 1a). Whereas only one cell line was homozygous for *MICA-129Met*, nine cell lines were heterozygous and six cell lines homozygous for the *MICA-129Val* allele. Cell lines carrying the *MICA-129Val/Val* genotype showed a trend to have higher pmMICA expression intensities than those carrying one or two *MICA-129Met* alleles (*P* = 0.0699, *t* test) (Fig. 1d). To determine the effect of the MICA-129 dimorphism on shedding of MICA, the amount of sMICA in the supernatant of the cell lines cultured for 24 h was determined in parallel (Fig. 1b). The expression of pmMICA and release of sMICA did not strictly correlate. Hela cells, having on average the highest amounts of pmMICA, hardly released any sMICA. However, overall *MICA-129Val* homozygous cell lines appeared to release more sMICA although this trend was not statistically significant (*P* = 0.1986, *t* test) (Fig. 1e). Cell lines carrying the *MICA-129Val/Val* genotype appeared to have higher amounts of intracellular MICA (icMICA) as determined by ELISA (Fig. 1c) than those carrying one or two *MICA-129Met* alleles (Fig. 1f, borderline significant *P* = 0.0777, *t* test).

**Fig. 2:**
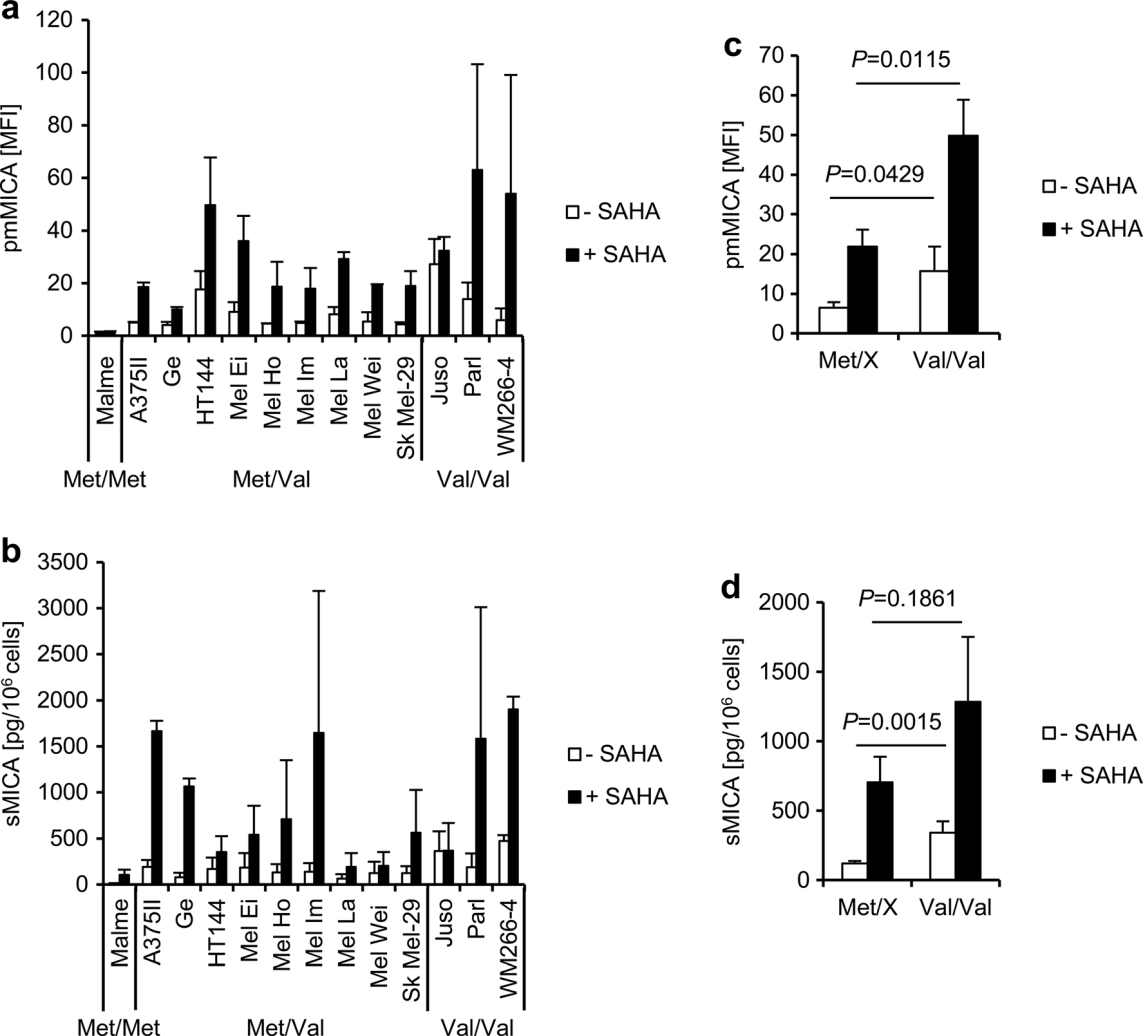
MICA expression on the plasma membrane and release of sMICA in human melanoma cell lines with different *MICA-129* genotypes. **a** The pmMICA expression of human melanoma cell lines carrying the different *MICA-129* genotypes (Met/Met, Met/Val, and Val/Val) was analyzed by flow cytometry in control cells (*−SAHA*) and cells treated with the HDAC inhibitor SAHA (10 μM) for 20 h before analysis (*+SAHA*). The MFI of pmMICA is shown as means plus SD (*n* ≥ 3). **b** In parallel, the amounts of soluble MICA (*sMICA*) in the supernatants were determined by ELISA and are shown as means plus SD of sMICA (pg/10^6^ cells). **d**, **e** The data were grouped according to the *MICA-129* genotype, and cell lines carrying one or two *MICA-129Met* alleles (Met/X, i.e., Met/Met and Met/Val) were compared to cell lines which were homozygous for the *MICA-129Val* allele. The data are displayed as means plus SEM, and they were analyzed by *t* tests

Since the variations in MICA expression and release were high in this panel of tumor cell lines of different entities, we next investigated the MICA expression in a collection of 13 melanoma cell lines. One melanoma cell line was homozygous for *MICA-129Met*, nine were heterozygous, and three were homozygous for the *MICA-129Val* allele (Fig. 2a). The MICA plasma membrane expression intensity was on average significantly higher in the melanoma cell lines carrying a *MICA-129Val/Val* genotype than in those which carried one or two *MICA-129Met* alleles (*P* = 0.0429, *t* test) (Fig. 2c). Since the MICA expression was in general low on the melanoma cell lines, we induced MICA expression by adding the HDAC inhibitor SAHA for 20 h to the cell culture medium (Elsner et al. 2010; Elsner et al. 2007; Skov et al. 2005) (Fig. 2a). The cell surface expression of MICA increased in all melanoma cell lines except Malme, which remained negative for pmMICA even after treatment with SAHA (Fig. 2a). The density of pmMICA was again significantly higher on the *MICA-129Val* homozygous melanoma cell lines than on those which carried one or two *MICA-129Met* alleles (*P* = 0.0115, *t* test) (Fig. 2c). The spontaneous release of sMICA was low in all melanoma cell lines but increased after treatment with SAHA (Fig. 2b). Melanoma cell lines carrying a *MICA-129Val/Val* genotype released significantly more sMICA than those which carried one or two *MICA-129Met* alleles (*P* = 0.0015, *t* test) (Fig. 2d). However, after SAHA treatment, the difference in the amounts of sMICA did not reach statistical significance (*P* = 0.1861, *t* test) (Fig. 2d).

### Expression of the MICA-129Met/Val isoforms in the MICA-deficient human melanoma cell line Malme

**Fig. 3:**
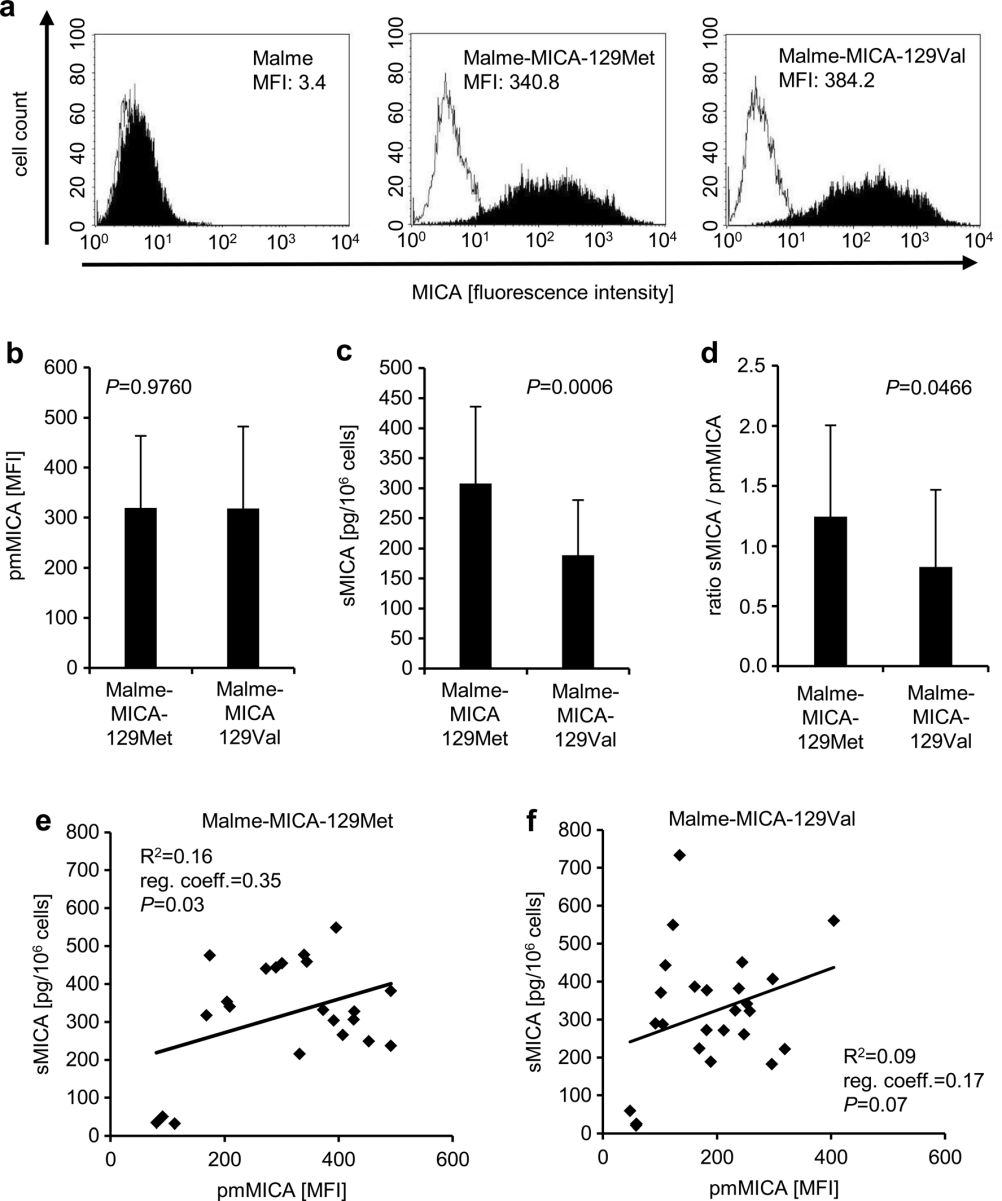
MICA shedding in Malme cells expressing the MICA-129Met or MICA-129Val variants. **a** The pmMICA expression intensity was determined by flow cytometry as exemplified for a Malme-MICA-129Met and a Malme-MICA-129Val clone and compared to parental Malme cells. The *white histograms* indicate cells stained with the secondary Ab only; the *black histograms* show the staining with anti-MICA plus secondary Ab. **b** A summary of pmMICA expression intensity (mean + SD) on Malme-MICA-129Met (*n* = 22) and Malme-MICA-129Val clones (*n* = 25) is displayed. **c** In parallel, the amount of sMICA in the supernatant was determined by ELISA (pg/10^6^ cells). **d** The ratios of sMICA/pmMICA were calculated. The data in **b**, **c**, and **d** were compared by a *t* test. **e** The linear regressions of sMICA and pmMICA were determined for the Malme-MICA-129Met and **f** Malme-MICA-129Val clones. The coefficient of determination (*R* ^2^), the regression coefficient (*reg. coeff.*), which is slope of the regression line, and the *P* value (for the Pearson correlation) are indicated for both MICA variants

To clarify whether MICA shedding is directly affected by the *MICA-129* genotype, the pmMICA-deficient melanoma cell line Malme was selected for further experiments. We had previously cloned the MICA*00701 allele, which contains a methionine (Met) at amino acid position 129 (pCMV6-AC-MICA-129Met), and generated the MICA-129Val variant of the MICA*00701 allele, which contains a valine (Val) at this position (pCMV6-AC-MICA-129Val) by site-directed mutagenesis (Isernhagen et al. 2015). Malme cells were transfected with these expression constructs and several clones expressing the MICA-129Met or MICA-129Val variants obtained by limiting dilution. Representative flow cytometry histograms for Malme wild-type cells and clones expressing the MICA-129Met and MICA-129Val variant are displayed in Fig. 3a. A broad range of pmMICA expression intensities were observed within (Fig. 3a) and between different clones (Fig. 3b) but on average, the expression was similar on Malme-MICA-129Met and Malme-MICA-129Val clones (*P* = 0.9760, *t* test).

### Effect of the MICA-129 dimorphism on shedding of MICA

The shedding of MICA was then analyzed in parallel to pmMICA expression intensity of MICA in these clones. Notably, the amount of sMICA in the supernatant of these cells (Fig. 3c) was higher for cells transfected with the MICA-129Met variant (*P* = 0.0006, *t* test). Accordingly, also the ratio of sMICA and pmMICA expressions (Fig. 3d) was higher for the Malme-MICA-129Met than Malme-MICA-129Val clones (*P* = 0.046, *t* test). Moreover, the amount of sMICA in the supernatant was partly dependent on pmMICA expression intensity for Malme-MICA-129Met cells as indicated by the coefficient of determination (*R*^2^ = 0.16) (Fig. 3e) but even less for Malme-MICA-129Val cells (*R*^2^ = 0.09) (Fig. 3f). These results suggested that the MICA-129Met/Val dimorphism directly influences MICA shedding and that the MICA-129Met variant is more susceptible to shedding than the MICA-129Val isoform. This result was not expected in view of the data obtained from non-transgenic tumor cell lines, in which we observed a higher pmMICA expression and a higher release of sMICA in cell lines with the *MICA-129Val/Val* genotype. Therefore, we speculated that the polymorphism could also directly affect pmMICA expression intensity and thereby control the release of sMICA.

### The MICA-129 dimorphism affects the MICA expression at the plasma membrane

**Fig. 4:**
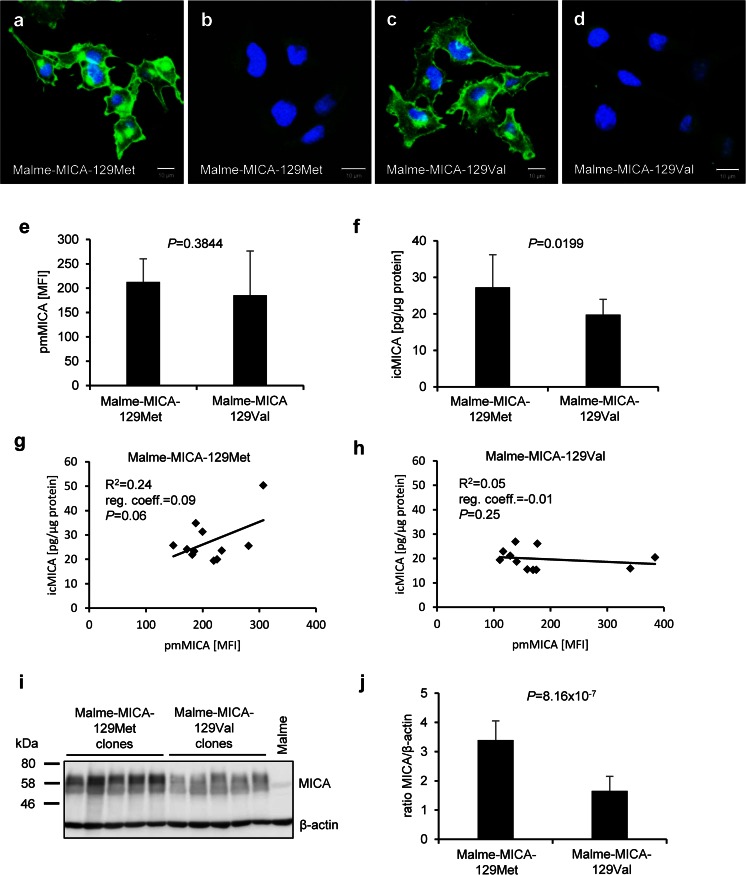
MICA expression on the plasma membrane and in intracellular compartments of Malme cells expressing the MICA-129Met or MICA-129Val isoform. **a** Confocal microscopy images show pmMICA and icMICA in a Malme-MICA-129Met clone after staining with an anti-MICA plus secondary Ab (*green*). Nuclei are stained with Hoechst 33342 (*blue*). The *scale bar* represents 10 μm. **b** The control illustrates the staining with the secondary Ab only. **c** A Malme-MICA-129Val clone is displayed after staining with an anti-MICA plus secondary Ab (*green*). **d** The control illustrates the staining with the secondary Ab only. **e** The pmMICA expression intensity on Malme-MICA-129Met (*n* = 11) and Malme-MICA-129Val clones (*n* = 11) is summarized as mean plus SD. The data were compared by a *t* test. **f** In parallel, the amounts of intracellular MICA (*icMICA*) were determined by ELISA and are shown as means plus SD. The data were compared by a *t* test. The linear regressions of icMICA and pmMICA are displayed for the **g** Malme-MICA-129Met and **h** Malme-MICA-129Val clones. The coefficient of determination (*R* ^2^), the regression coefficient (*reg. coeff.*), and the *P* value (for the Pearson correlation) are indicated for both MICA variants. **i** An immunoblot is shown on which lysates of five Malme-MICA-129Met clones, five Malme-MICA-129Val clones, and parental Malme cells were probed with anti-MICA and, as loading control, anti-β-actin Abs. The bands of a protein size marker are indicated at the *left* side. MICA bands are broad due to variations of glycosylation. **j** Immunoblots were analyzed by densitometry, and a summary of MICA/β-actin ratios of Malme-MICA-129Met (*n* = 11) and Malme-MICA-129Val (*n* = 11) clones is displayed as means plus SD. The data were compared by a *t* test

Immunofluorescence staining of MICA was performed to compare the subcellular expression pattern of the MICA-129Met and the MICA-129Val isoforms in the Malme clones. Both MICA variants were found on the plasma membrane but also in intracellular compartments. A larger proportion of the MICA-129Met than the MICA-129Val variant appeared to be localized intracellularly (Fig. 4a–d). To quantify this distribution, we measured in parallel pmMICA by flow cytometry (Fig. 4e) and icMICA in cellular lysates by ELISA (Fig. 4f) and Western blot (Fig. 4j) in 11 clones of each genotype. Again, the expression of pmMICA was overall similar in Malme-129Met and Malme-129Val clones (*P* = 0.3844, *t* test) (Fig. 4e). However, significantly more icMICA was found in the Malme-129Met than in Malme-129Val clones by ELISA (*P* = 0.0199, *t* test) (Fig. 4f). Notably, the amount of icMICA increased with the pmMICA expression intensity for Malme-MICA-129Met clones (regression coefficient = 0.09, *R*^2^ = 0.24) (Fig. 4g) but not for Malme-MICA-129Val clones (regression coefficient = −0.01, *R*^2^ = 0.05) (Fig. 4h). Furthermore, cellular lysates of these clones were tested by Western blot for MICA expression as illustrated in Fig. 4i. Densitometry of MICA in comparison to β-actin (Fig. 4j) indicated a higher ratio of MICA/β-actin in Malme-MICA-129Met than in Malme-MICA-129Val clones (*P* = 8.16 × 10^−7^). Together, these results indicated that a larger proportion of the MICA-129Met than the MICA-129Val variant was localized intracellularly.

**Fig. 5:**
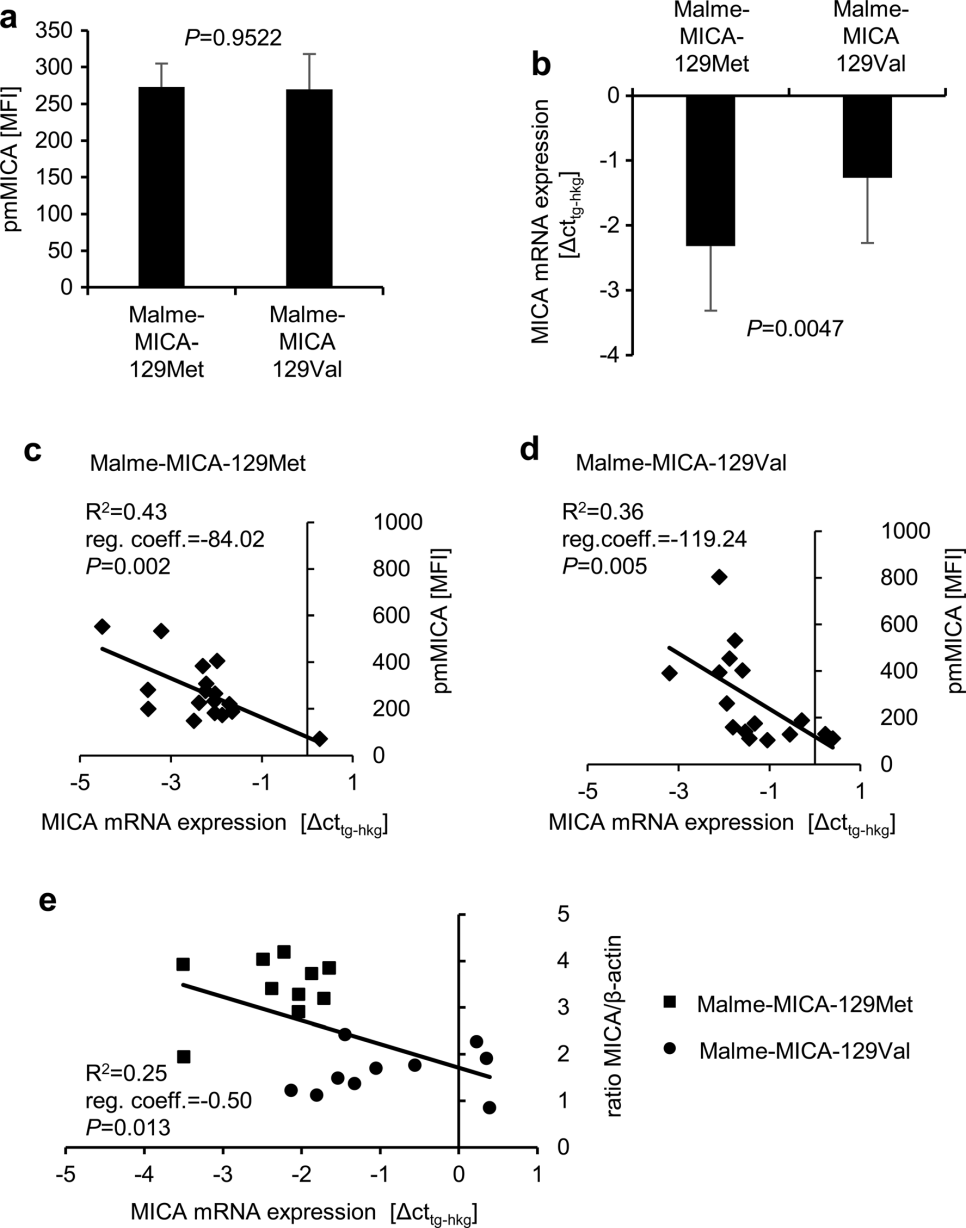
Correlation of *MICA* mRNA and pmMICA expression intensity in Malme cells expressing the MICA-129Met and MICA-129Val variants. **a** The MFI of pmMICA expression was determined by flow cytometry on Malme-MICA-129Met (*n* = 17) and Malme-MICA-129Val (*n* = 17) clones. The data are summarized as means plus SD and were compared by a *t* test. **b** In parallel, the *MICA* mRNA expression (ΔCt_tg−hkg_) was determined by qPCR. Expression values of the target gene (t*g*) *MICA* were calculated from means of technical triplicates after normalization to the housekeeping gene (*hkg*) *GAPDH*. The data are summarized as means plus SD and were compared by a *t* test. It should be noted that lower ΔCt_tg−hkg_ values indicate higher mRNA expression levels. The linear regressions of *MICA* mRNA expression and pmMICA expression are displayed for **c** Malme-MICA-129Met and **d** Malme-MICA-129Val clones. The coefficient of determination (*R* ^2^), the regression coefficient (*reg. coeff.*), and the *P* value (for the Pearson correlation) are indicated for both MICA variants. **e** The linear regression of *MICA* mRNA expression and total MICA expression as determined by Western blot is shown for Malme-MICA-129Met and Malme-MICA-129Val clones, in which mRNA and protein expression had been measured in parallel

We then compared in parallel *MICA* mRNA and pmMICA expression in 17 Malme-129Met and 17 Malme-129Val clones. The pmMICA expression was again not significantly different in these experiments (*P* = 0.9522, *t* test) (Fig. 5a), but more *MICA* mRNA was found in the Malme-129Met than Malme-129Val clones (*P* = 0.0047, *t* test) as indicated by lower Δct values (Fig. 5b). In both Malme-MICA-129Met and Malme-MICA-129Val clones, the *MICA* mRNA expression intensity correlated with the pmMICA expression intensity (*R*^2^ = 0.43 for Malme-MICA-129Met and *R*^2^ = 0.36 for Malme-MICA-129Val clones) (Fig. 5c, d). Overall, the MICA mRNA expression correlated also with total MICA protein expression as determined by MICA/β-actin ratio in Western blot (*R*^2^ = 0.25). However, since the clones had been selected for similar pmMICA expression intensities, both MICA mRNA and protein expression was higher for Malme-MICA-129Met than Malme-MICA-129Val clones (Fig. 5e).

**Fig. 6:**
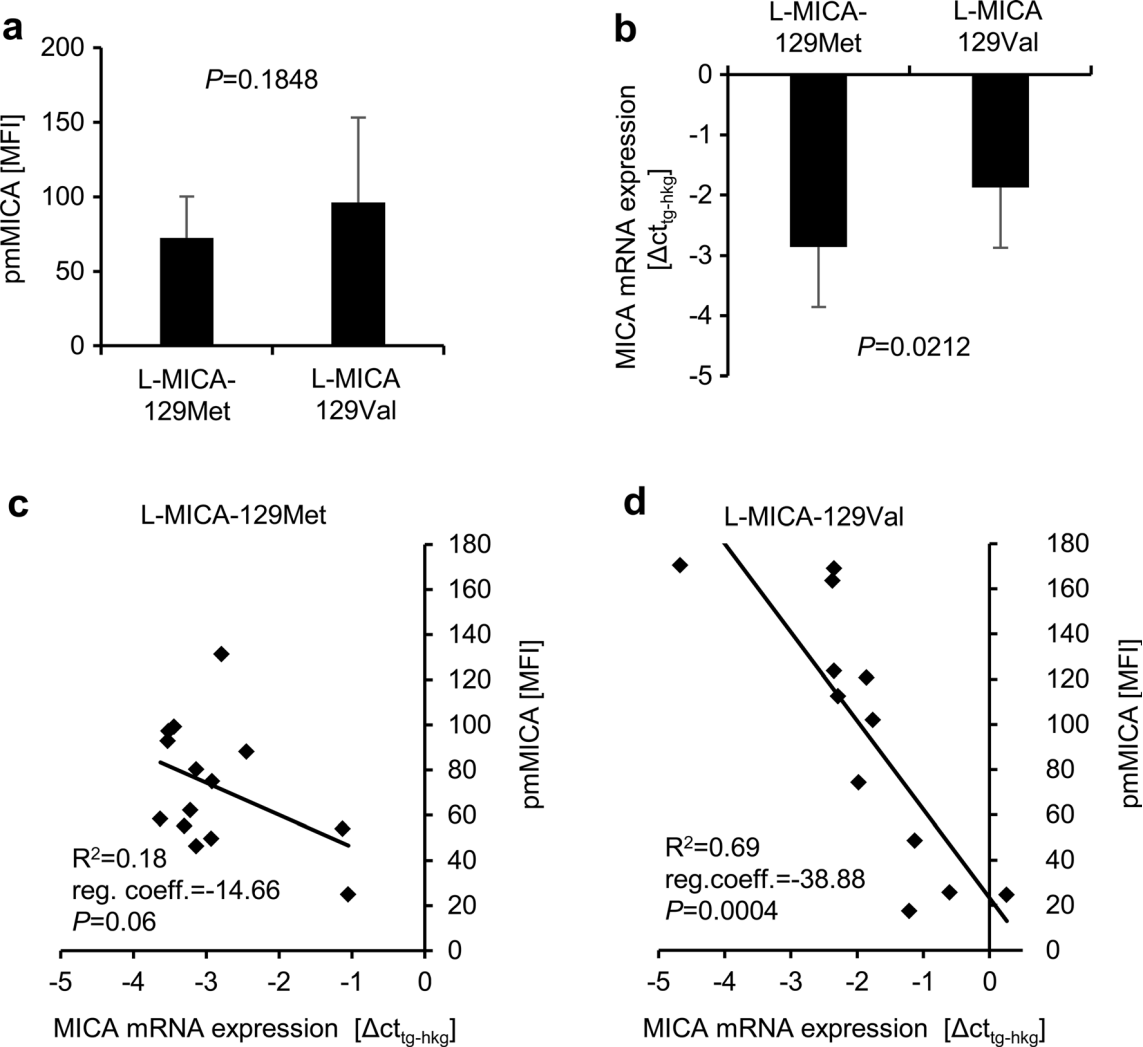
Correlation of *MICA* mRNA and pmMICA expression intensity in L cells expressing the MICA-129Met and MICA-129Val variants. **a** The MFI of pmMICA expression was determined by flow cytometry on L-MICA-129Met (*n* = 14) and L-MICA-129Val (*n* = 12) clones. The data are summarized as means plus SD and were compared by a *t* test. **b** In parallel, the *MICA* mRNA expression (ΔCt_tg−hkg_) was determined by qPCR. Expression values of the target gene (*tg*) *MICA* were calculated from mean of technical triplicates after normalization to the housekeeping gene (*hkg*) *Hprt*. The data are summarized as means plus SD and were compared by a *t* test. It should be noted that lower ΔCt_tg−hkg_ values indicate higher mRNA expression levels. The linear regressions of *MICA* mRNA expression and pmMICA expression are displayed for **c** L-MICA-129Met and **d** L-MICA-129Val clones. The coefficient of determination (*R* ^2^), the regression coefficient (*reg. coeff.*), and the *P* value (for the Pearson correlation) are indicated for both MICA variants

To confirm the results obtained with the Malme clones, we also analyzed mouse fibroblast L cells, which previously had been transfected with the pCMV6-AC-MICA-129Met/Val expression constructs (Isernhagen et al. 2015). Murine cells do not have a *MICA* gene and from these L cells MICA was not released by shedding (Isernhagen et al. 2015). The MICA cell membrane expression of the L cell clones was on average similar between L-MICA-129Met and L-MICA-129Val clones (Fig. 6a) (*P* = 0.1848, *t* test). In line with the results obtained with Malme clones, the *MICA* mRNA expression (Fig. 6b) was also significantly higher (indicated by lower Δct values) in L cell clones expressing the MICA-129Met than the MICA-129Val variant (*P* = 0.0212, *t* test). The pmMICA expression intensity was weakly dependent on the mRNA expression intensity in clones transfected with the MICA-129Met variant as indicated by the coefficient of determination (*R*^2^ = 0.18) (Fig. 6c). However, for L cells expressing the MICA-129Val variant, the *MICA* mRNA and pmMICA expression intensities correlated tightly (*R*^2^ = 0.69) (Fig. 6d).

Taken together, the MICA-129Met/Val dimorphism appears to affect MICA plasma membrane expression intensity and shedding, and both processes together determine the extent of sMICA release.

## Discussion

Numerous studies have identified *MICA* polymorphisms to be associated with various malignant and autoimmune diseases. In view of the biological function of MICA as a NKG2D ligand, these data suggest that *MICA* variants themselves could be causative for at least some of these associations despite linkage disequilibrium in the HLA region. The SNP (rs1051792) of the *MICA* gene resulting in the MICA-129Met/Val dimorphism was the first *MICA* polymorphism for which a functional consequence was described. Steinle and colleagues identified the MICA-129Met variants as high and MICA-129Val variants as low avidity NKG2D ligands (Steinle et al. 2001). Recently, we investigated the functional consequences of this polymorphism in more detail (Isernhagen et al. 2015). Engagement of NKG2D on NK cells by the high-avidity MICA-129Met variant was characterized by stronger and faster NKG2D signaling resulting in more NK cell cytotoxicity and interferon-γ release. On CD8^+^ T cells, the MICA-129Met variant mediated a faster co-stimulation and activated the cells in combination with limited CD3-mediated signals. Notably, on both cell types, the MICA-129Met variant induced also a rapid NKG2D downregulation. Therefore, the effects elicited by the MICA-129Met variant were not sustained and at high MICA expression intensities, the MICA-129Val variant elicited even more NKG2D-mediated responses, such as NK cell cytotoxicity, than the MICA-129Met variant.

Two recent studies suggested that the MICA-129Val/Val genotype was associated with higher sMICA serum levels in patients with ulcerative colitis (Zhao et al. 2011) as well as in patients with hepatitis B virus-induced hepatocellular carcinoma and healthy controls (Tong et al. 2013). It was unclear, however, whether the MICA-129 dimorphism has a direct effect on the generation of sMICA or is linked to other polymorphisms affecting MICA shedding. We were therefore interested to clarify whether the MICA-129Met/Val dimorphism not only directly affects NKG2D signaling but also MICA shedding.

We investigated pmMICA expression in parallel to release of sMICA in 16 tumor cell lines of various entities and 13 melanoma cell lines. The MICA plasma membrane expression intensities on the melanoma cell lines were higher on cell lines, which were homozygous for the MICA-129Val allele than on those which carried either one or two MICA-129Met alleles. Similarly, the amount of sMICA released from the melanoma cell lines carrying two MICA-129Val alleles was higher than from those which carried one or two MICA-129Met alleles. The panel of various tumor cell lines displayed the same trends. The two cell lines, which were homozygous for the MICA-129Met variant (Malme, melanoma and T47D, breast cancer), were both negative for pmMICA expression and released very little sMICA into the cell culture supernatant. The plasma membrane expression was not (Malme) or only slightly (T47D, data not shown) inducible by treatment with the HDAC inhibitor SAHA. Although these results suggested that the expression of MICA-129Met variants at the plasma membrane is lower and that MICA-129Met variants are less released by shedding than MICA-129Val variants, the number especially of *MICA-129Met* homozygous cell lines has been too low to draw definitive conclusions since *MICA* alleles can vary with respect to mRNA expression intensity (Shafi et al. 2011). Notably, some *MICA* alleles such as MICA*008 can have various promoter variants (Cox et al. 2014), which might alter expression intensities.

Therefore, we transfected the MICA-negative melanoma cell line Malme with expression constructs for MICA which differed only at position 129 and obtained cell clones expressing both MICA variants at similar intensities. Malme-MICA-129Met clones released more sMICA than Malme-MICA-129Val clones. This finding indicates that the MICA-129Met/Val dimorphism does affect MICA shedding. The MICA-129Met/Val dimorphism is localized in the α2 domain, which is far away from the cleaving site in the stalk region. Therefore, it influences shedding likely indirectly by a conformational change affecting the accessibility of MICA for ADAM family proteases or the chaperone ERp5.

In contrast to this result, we found more sMICA in the supernatant of tumor and melanoma cell lines carrying a *MICA-129Val/Val* genotype. Notably, these cell lines expressed also more MICA on their plasma membrane. Therefore, we compared the expression pattern of MICA in the Malme transfectants. Both MICA variants were found at the plasma membrane in similar amounts, but in intracellular compartments, more MICA was found in Malme cells expressing the MICA-129Met than the MICA-129Val variant. This result suggests that the MICA-129Met and MICA-129Val variants either differ in the efficacy of transport to the cell surface, in recycling into intracellular compartment after expression at the plasma membrane, or in degradation after internalization. An alteration of the intracellular transport has been described previously for *MICA-A5.1* variants (Ashiru et al. 2013). Moreover, it has been recently reported that the N glycosylation of asparagine 8 in MICA018 is important for cell surface expression of this variant (Mellergaard et al. 2014), but the MICA-129Met/Val dimorphism has previously not been implicated in the regulation of cell surface expression.

As mentioned before, the cell clones were selected to have on average a similar pmMICA expression intensity. Nonetheless, the selected Malme-MICA-129Met clones expressed more *MICA* mRNA than Malme-MICA-129Val clones. Similarly, in mouse L cells which were transfected with the same constructs, more *MICA-129Met* than *MICA-129Val* mRNA was expressed in clones which had similar pmMICA expression intensities. The correlation of *MICA* mRNA and pmMICA expression appeared to be partly better for mouse L cells than for human MALME cells. This could be due to the failure of L cells to shed MICA (Isernhagen et al. 2015). Proteins involved in shedding of NKG2D ligands might not be expressed in L cells or fail to interact with human MICA. Moreover, other proteins which retain MICA-129Met variants in human cells could not be present or fail to interact with MICA in mouse cells.

Taken together, these results show that the MICA-129Met variant was less efficiently expressed at the plasma membrane than the MICA-129Val variant. A larger proportion of the MICA-129Met variant was in intracellular compartments. Whether this was due to alteration of transport, recycling or degradation remains to be elucidated. In Malme and L cells, more mRNA of the MICA-129Met than the MICA-129Val variant was required to obtain a similar pmMICA expression intensity.

The generation of sMICA can be affected by polymorphisms, which regulate cell surface expression, and polymorphisms, which alter the efficacy of cleavage by shedding proteases. Our results demonstrate that the MICA-129 dimorphism affects both processes leading to a reduced cell surface expression and an increased shedding of MICA-129Met variants. Both processes limit the cell surface expression of the high-avidity MICA-129Met variant, which causes a strong NKG2D counter-regulation, if present at high density and for prolonged time (Isernhagen et al. 2015). On the other hand, even low amounts of MICA-129Met signals can activate a strong and fast NKG2D signaling (Isernhagen et al. 2015). Therefore, both MICA-129 variants may confer advantages and disadvantages in certain situations, such as virus infections, suggesting balancing evolution of *MICA* alleles. The mechanisms by which the MICA-129 dimorphism affects plasma membrane expression and shedding need to be investigated further. Although the MICA-129Val variant was apparently less efficiently cleaved to produce sMICA, the higher plasma membrane expression intensity of MICA-129Val variants could explain the association of this variant with higher sMICA concentration in tumor cell culture supernatants as shown here or in patient sera as described previously by others (Tong et al. 2013; Zhao et al. 2011).

In conclusion, we have shown that the MICA-129 dimorphism directly affects plasma membrane expression and shedding and these functional effects might contribute to the numerous disease associations, which have been reported for this polymorphism.
